# Cerebral gray matter volume correlates with fatigue and varies between desk workers and non-desk workers

**DOI:** 10.3389/fnbeh.2022.951754

**Published:** 2022-09-15

**Authors:** Handityo A. Putra, Kaechang Park, Fumio Yamashita, Yoshinori Nakagawa, Toshiya Murai

**Affiliations:** ^1^Department of Engineering, Keimyung University, Daegu, South Korea; ^2^Traffic-Medicine Laboratory, Kochi University of Technology, Kami, Japan; ^3^Division of Ultrahigh Field MRI, Institute for Biomedical Sciences, Iwate Medical University, Morioka, Japan; ^4^Department of Management, Kochi University of Technology, Kami, Japan; ^5^Department of Psychiatry, Faculty of Medicine, Kyoto University, Kyoto, Japan

**Keywords:** chronic fatigue syndrome, regional gray matter volume, *Karoshi*, chalder Fatigue Questionnaire, voxel-based morphometry (VBM)

## Abstract

Chronic fatigue syndrome (CFS), a clinical entity of chronic fatigue, has been associated with a decrease in regional gray matter volume (rGMV). In this study targeting a large number of healthy middle-aged individuals without CFS, the relationship between fatigue perception and rGMV was investigated. Considering that the work setting is an environmental factor that influences fatigue perception among healthy individuals, the differences between desk workers and non-desk workers were investigated. Chalder Fatigue Questionnaire (CFQ) scores were used for perceptional evaluation of fatigue, and rGMV of 110 brain regions was adapted with Statistical Parametric Mapping (SPM) 8 on 1.5 T magnetic resonance imaging (MRI) results for the volumetric calculation of gray matter. The CFQ scores were negatively correlated with the right supplementary motor area (SMC) and positively correlated with the right superior parietal lobule (SPL) and left basal forebrain in all participants (*n* = 1,618). In desk workers and non-desk workers, the CFQ scores correlated with different regions and yielded different mechanisms of fatigue perception in the brain. Identifying the gray matter regions correlated with fatigue perception in healthy individuals may help understand the early stage of fatigue progression and establish future preventive measures.

## Introduction

Fatigue, along with fever and pain, are the three major symptoms reported in patients with common diseases. Chronic fatigue (CF), a persistent type of perceived fatigue, often causes physical exhaustion, and mental health issues such as depression (Okada et al., 2004). The Japanese word *karoshi*, directly translated to “overwork death,” has been described as mortality caused by CF (Kanai, 2009). Chronic fatigue syndrome (CFS) is a chronic disease characterized by severe fatigue of unknown origin, which continues for 6 months or more, also referred to as myalgic encephalomyelitis or post-viral fatigue syndrome (Cope and David, 1996; Griffith and Zarrouf, 2008). CF and CFS, including *karoshi*, are well-known national health management challenges to overcome, especially in Japan (Kondo and Oh, 2010). Early understanding and identification of fatigue before it becomes chronic is essential as a preventive strategy for CF and CFS.

Further, an effective understanding of fatigue development may result in the establishment of CF and CFS countermeasures. Thus, fatigue studies should target adults without CF or CFS in the early stages of fatigue development. In particular, middle-aged adults are the ideal population to elucidate this mechanism because they comprise the majority of *karoshi* victims (Kondo and Oh, 2010). The degree of fatigue is also directly related to overwork, which is seen more frequently in middle-aged individuals than in younger individuals (Kanai, 2009; Kondo and Oh, 2010). Middle-aged individuals work in various settings and experience varying degrees of perceived fatigue. In recent years, work types have been characterized by hours spent sitting at a desk because longer sitting hours in a day are associated with higher morbidity and mortality (Bauman et al., 2011; van der Ploeg et al., 2012). Therefore, in this study, we examined healthy middle-aged individuals (45–65 years of age), who were classified by work setting based on sitting hours to elucidate the early signs and deterioration or chronicity of perceived fatigue. This study needed a large number of participants because of the classification based on age range and work types.

Previous studies on the relationship between the brain and fatigue have been performed on patients with CFS (Okada et al., 2004; de Lange et al., 2005; Barnden et al., 2011; Tang et al., 2015; Finkelmeyer et al., 2018). Most of these studies were based on functional images and the metabolism of the brain (Derache et al., 2013), whereas gray matter (GM) and white matter (WM) volumetric studies for CFS were scarce. An early voxel-based morphometry (VBM) study reported a reduction of GM volume in the bilateral prefrontal cortex, which was correlated with reduced functional status (Okada et al., 2004). Another VBM study showed regional GM volume reductions only in the occipital and para-hippocampal regions, and WM volume reductions in the occipital areas (Puri et al., 2012). However, an overall decrease in WM volume and no difference in GM volume has been reported (Zeineh et al., 2015).

On the other hand, overall decreases in GM volume have been reported in two independent cohorts of CFS patients (de Lange et al., 2005), but these results were not replicated in the subsequent study by the same group (van der Schaaf et al., 2017) or in recent studies by other groups (Barnden et al., 2011, 2016). All studies related to the cohorts found no significant differences in the rGMV or WM volumes of patients with CFS and healthy controls. Nevertheless, a more recent study showed that the amygdala and insula, related to the processing of interoceptive signals and stress, in CFS patients did not decrease but increased in volume. Further, reductions in WM volume were identified in the midbrain, pons, and right temporal lobe (de Lange et al., 2005). Thus, there may be a more complicated reaction of brain volume to fatigue development, although the patient sample sizes of all previous studies were not so large as to yield reproducible findings.

There has been only one cross-sectional fatigue study with healthy individuals that examined more than 800 young adults with a mean age of 20.7 years [standard deviation (*SD*), 1.81] (Nakagawa et al., 2016). The study reported no significant correlations between volume differences in rGMV and WM based on the degree of fatigue. However, diffusion tensor imaging (DTI) showed that the mean diffusivity in the right putamen, right pallidus, and right caudate were significantly associated with the degree of fatigue and motivation, whereas physical activity evaluated by the questionnaire was significantly correlated with the right putamen. The authors presented a plausible mechanism of fatigue, which involve abnormal function of the motor system and the dopaminergic system in the basal ganglia. Nevertheless, young patients are not as prone to suffer from severe fatigue, e.g., *karoshi*. Furthermore, work types and lifestyles affecting the degree of fatigue were not considered in the study.

For this study, we gathered 1,618 middle-aged adults who fully satisfied the inclusion criteria, and were divided by work setting. In order to carry out this study design, we examined nearly 3,000 patients undergoing brain health checkups, which have been uniquely developed and are a prevalent preventive healthcare strategy in Japan.

## Materials and methods

### Participants and work types

**FIGURE 1:**
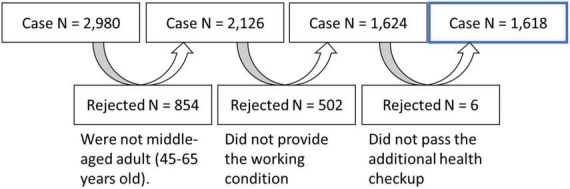
Participant selection process. The flowchart depicts the reduction in sample size after vetting through inclusion criteria.

At the Kochi Kenshin Clinic attached to the Kochi University of Technology, a total of 2,980 people (20–89 years old) underwent magnetic resonance imaging (MRI) examinations and answered fatigue questionnaires for brain health examinations. Written informed consent was obtained from each participant for the projects in which they participated. The procedures for all studies were conducted in accordance with the Declaration of Helsinki and approved by the Ethics Committee of the Kochi University of Technology. All images went through a series of visual quality checks to check for space-occupying lesions such as brain tumors and arachnoid cysts. Since this study focused on middle-aged adults between the ages of 45 and 65, 854 cases were excluded. The remaining middle-aged adults were interviewed and asked about their type of work and how long they sit each day, and a further 502 individuals were excluded from the study because they could not provide sufficient information regarding their work setting. In addition, six cases were excluded as the patients were later diagnosed with cerebrovascular diseases such as stroke. Finally, 1,618 individuals (mean age: 52.89, *SD*: 4.59) participated in the study and were asked to complete the Chalder Fatigue Questionnaire (CFQ). The participants’ selection process is illustrated in Figure 1, and the participants’ demographic data are presented in Table 1.

**TABLE 1:** Differences in age, CFQ score, and several brain region volumes (divided and standardized by intracranial volume) between work type groups.

	Desk workers		Non-desk workers		
Measures	*N* = 942		*N* = 676		*t*-test (*P*-value)
	Mean	(*SD*)	Mean	(*SD*)	
Age	52.668	(4.607)	52.973	(4.546)	0.933
CFQ	25.787	(5.806)	25.635	(5.777)	0.730
Total BV/ICV	0.8250	(0.0175)	0.8259	(0.0167)	0.090
Total GMV/ICV	0.4275	(0.0182)	0.4277	(0.0181)	0.975
Total WMV/ICV	0.3975	(0.0177)	0.3981	(0.0174)	0.737

CFQ, the Chalder’s fatigue questionnaire; BV, brain volume; GMV, gray matter volume; WMV, white matter volume; ICV, intra-cranial volume; SD, standard deviation.

Work settings were classified into two types: desk type, where the sitting hours were more than half of the total working hours; and non-desk type, where the sitting hours were less than half of the working hours. The participants worked for 8–10 h a day; thus, participants sitting more than 4 h daily in the workplace on average were included within the desk type group. For example, the desk type group exclusively included local public officials, office workers, teachers, IT engineers, doctors, and directors. The non-desk type group included individuals performing excessive physical movements, such as physical laborers, field workers, nurses, and nursery schoolteachers.

### Fatigue assessment

The degree of fatigue was evaluated using the CFQ (Chalder et al., 1993), a self-administered questionnaire that quantifies the extent and severity of subjective fatigue within both clinical and non-clinical populations. Although initially developed to measure the degree of chronic fatigue symptoms within clinical populations (Deale et al., 1997), the scale was revised and is now widely used to measure the severity of “tiredness” in a non-clinical, general population (Cella and Chalder, 2010; Chilcot et al., 2016).

The questions are benign and non-threatening, asking about sensation and functionality. Each of the 11 items is answered on a 4-point scale ranging from asymptomatic to maximum symptomologies, such as “Better than usual,” “No worse than usual,” “Worse than usual,” and “Much worse than usual.” For all items, the least symptomatic answers are on the left of the response set, providing an easy-to-understand checklist for respondents. The global score ranges from 0 to 33, with 0 indicating no fatigue at all, and 33 indicating the extreme fatigue score.

Reliability coefficients for the CFQ are high in the studies of CFS patients (Morriss et al., 1998) as well as in occupational and general population research. The CFQ has been used widely in studies investigating tiredness among working populations and consistently fares exceptionally well against other longer and multidimensional tools (De Vries et al., 2003). Another advantage is that the CFQ is used widely in occupation-specific research and allows for easy comparisons between studies and populations.

### Magnetic resonance imaging

T1-weighted MR images were acquired using a 1.5 T ECHELON Vega system (Hitachi, Tokyo, Japan) with a three-dimensional gradient-echo with inversion recovery (3D-GEIR) sequence. The following scanning parameters were used; repetition time, 9.2 ms; echo time, 4.0 ms; inversion time, 1,000 ms; flip angle, 8°; the field of view, 240 mm; matrix size, 0.9375 × 0.9375 mm; slice thickness, 1.2 mm; the number of excitations, 1. Each image was visually checked for brain disease and anomaly, as well as the head motion and artifacts that may affect the volumetric measurement. The images were processed and analyzed using the VBM8 toolbox^1^ and other modules implemented in Statistical Parametric Mapping (SPM) 8^2^ to estimate the regional brain volumes.

In brief, the images were segmented into gray matter, white matter, and cerebrospinal fluid (CSF) space using a maximum *a posteriori* (MAP) approach (Whitwell, 2009; Kurth et al., 2010). The segmented gray and white matter images were then used to estimate the morphological correspondence between the template image and the participant’s brain using the high-dimensional non-linear warping algorithm (Ashburner, 2007). The estimated non-linear warp was inversely applied to an atlas defined in the template space, to parcellate the target brain anatomically. The Neuromorphometrics atlas incorporated in SPM12 was used for percolation, with a modification for white matter lesions which appeared as incorrect gray matter segments around lateral ventricles. Volumes of each anatomical region were calculated as the sum of the corresponding tissue densities in the voxels belonging to each region.

### Statistical analysis

Three statistical analyses were performed in this study: bivariate correlation of all relevant parameters, a *t*-test between the desk work and non-desk workgroups, and multiple regression analyses to investigate rGMVs that affect CFQ scores. Correlation between fatigue score, work type, and brain volumes (total brain volume including GM, WM, and CSF volume, and rGMV in 110 regions of the brain) estimated by the SPM8 toolbox was investigated using bivariate correlation with SPSS version 22 (IBM Corp., Armonk, NY, United States).

**FIGURE 2:**
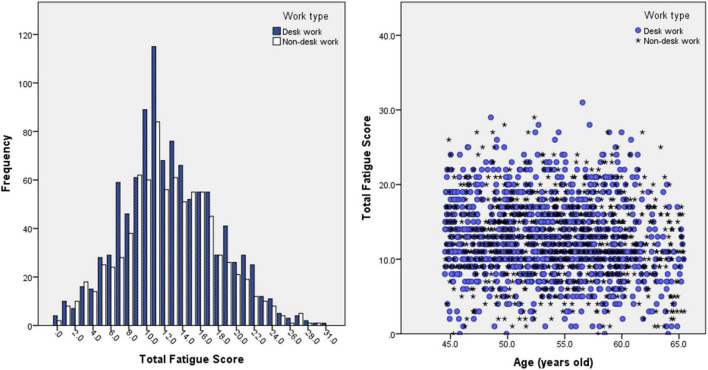
Histogram of CFQ (total fatigue score) result’s distribution for desk work and non-desk work participants (left) and scatter plot of CFQ score results vs. age. Both left and right figures show no significant and observable difference in CFQ scores between desk work and non-desk work participants.

The *t*-test between the desk work and non-desk workgroups was performed on all relevant parameters including, age, sex, CFQ score, and brain region volumes, in order to investigate whether the two groups have any correlations, similarities, or differences. Furthermore, an exploratory analysis of the relationship between segment volume or rGMV and fatigue was also performed using stepwise multiple linear regression analysis with 95% confidence intervals for each of the regional brain volumes. The stepwise multiple linear regression analysis was performed separately for each regional brain site and independently for each type of fatigue score. Table 2 shows the three groupings: All participants, desk work participants, and non-desk work participants. Age and sex (where the men are coded as 1 and women as 0) are added as covariate variables. The CFQ score distribution for each group is illustrated in Figure 2. The volume data used were the rGMVs that had already been divided by the brain’s intracranial volume (ICV). This strategy was chosen to minimize the variation in brain sizes among individuals.

**TABLE 2:** Linear regression (stepwise-forward) analysis results for all participants, desk workgroup, and non-desk workgroup, with CFQ score as the target variable and age, sex, work type, and volume of brain regions as the independent variables.

Parameters and brain regions that significantly correlates with CFQ scores		Coefficient		*T*-value
		B (slope)	Std. error	
All participants (*N* = 1,618)	Sex	–1.411	0.306	–4.614
	(1) Left basal forebrain	7.891	2.985	2.644
	(2) Right supplementary motor cortex	–1.495	0.473	–3.162
	(3) Right superior parietal lobule	0.668	0.258	2.592
Desk workers (*N* = 942)	Sex	–1.062	0.411	–2.585
	Age	–0.102	0.041	–2.482
	(4) Right putamen	1.840	0.589	3.124
	(5) Left frontal operculum	–4.305	1.546	–2.784
	(6) Left fusiform gyrus	1.283	0.534	2.403
	(7) Right occipital pole	–1.718	0.732	–2.349
	(2) Right supplementary motor cortex	–1.603	0.631	–2.541
Non-desk workers (*N* = 676)	Sex	–1.727	0.448	–3.856
	(8) Left anterior insula	6.182	1.669	3.705
	(9) Right anterior insula	–4.050	1.693	–2.392
	(10) Left lateral orbital gyrus	–4.312	1.845	–2.338

## Results

### Age, chalder fatigue questionnaire scores, and several brain volume data for work types

**FIGURE 3:**
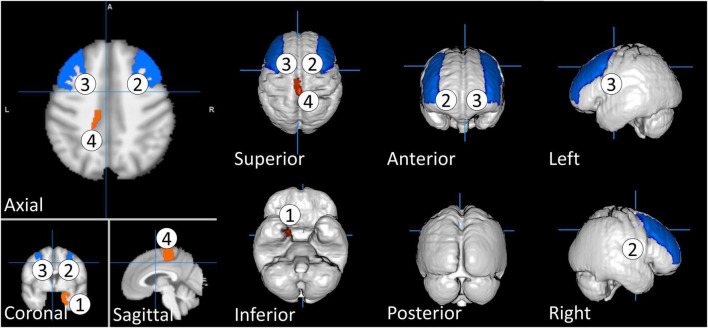
The regional gray matter volume that is significantly different between desk work and non-desk work participants. The regional gray matter volume is color-coded based on *t* values, with red indicating negative t value and blue indicating positive *t* value. We coded deskwork group as sample 1 and non-deskwork group as sample 2. The rGMVs are as follows: 1. Right entorhinal area, 2. Right middle frontal gyrus, 3. Left middle frontal gyrus, and 4. Left precentral gyrus medial segment.

Table 1 shows the mean and standard deviation characteristics of the study participants for age and CFQ, and brain regions including total brain volume (BV), total gray matter volume (GMV), and total white matter volume (WMV) divided by ICV. The CFQ score was found to be highly reliable (11 items; α = 0.89). Statistically, the *t*-test showed that there was no significant difference between desk work and non-desk work in terms of age, CFQ score, total brain volume, total GM volume, and total WM volume. The insignificance of the CFQ score indicates that participants in both groups perceived a similar level of fatigue, even though they work in different types of working environments. Therefore, for detailed rGMV, we further performed a *t*-test between the two groups to investigate whether there was a significant difference between participants in the desk work and the non-desk workgroups. The independent *t*-test analysis showed four rGMVs that were significantly different between the desk work and non-desk workgroups. In this test, deskwork is coded as 1 and non-deskwork as 2. The four rGMVs are as follows: right entorhinal area [*t*(1, 616) = −2.229, *p* = 0.026 < 0.05], right middle frontal gyrus [*t*(1, 616) = 2.268, *p* = 0.023 < 0.05], left middle frontal gyrus [*t*(1, 616) = 2.235, *p* = 0.026 < 0.05], and left precentral gyrus medial segment [*t*(1, 616) = −2.063, *p* = 0.39 < 0.05]. The rGMV that is significantly different between the desk and non-desk workgroups is illustrated and color-coded in Figure 3. These differences consider only the structural differences and not the effect on fatigue scores between the two groups. The results might indicate that although there is no significant difference in CFQ scores between the two groups, the impact of fatigue on their rGMVs is different.

### Analysis of white matter volume

We found no significant correlations between CFQ scores and WMV.

### Analysis of regional gray matter volume

**FIGURE 4:**
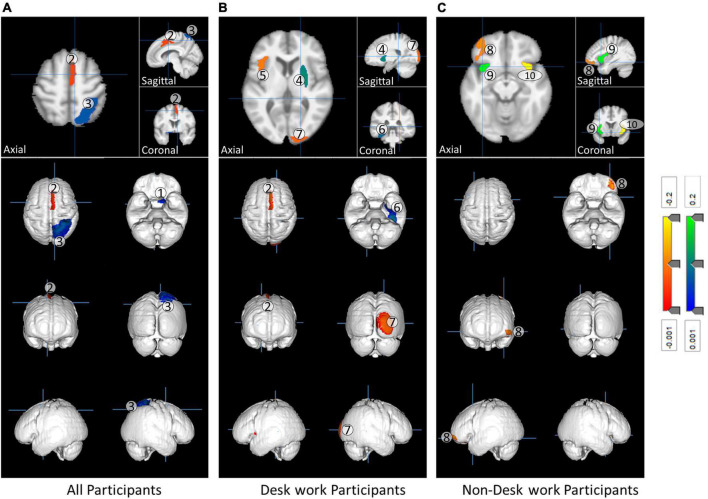
The impact of fatigue on gray matter volume in the participants. **(A)** All participants, **(B)** desk work participants, and **(C)** non-desk work participants. The most significant regions presented in Table 2 are color-coded based on Beta **(B)** values with red as a negative change and blue as a positive change. The rGMVs index in the figure follow the index presented in Table 2.

Three separate multiple linear regressions were performed on the data for participants in the desk, non-desk, and both desk and non-desk workgroups. The results of the analysis showed changes in GMV in several regions that were significantly associated with CFQ scores. Table 2 shows the brain regions that have a significant correlation between the CFQ score and rGMV, and Figure 4 illustrates them. The figure is color-coded in blue and red, showing negative and positive correlations, respectively. A positive correlation indicates that the affected rGMV has a higher CFQ score, and a negative correlation indicates that the volume of the affected rGMV has a lower CFQ score.

The partial regression plot and the studentized residual plot against the predicted values showed evidence of linearity in all three regression analyses. The independence of residuals for total, physical, and mental fatigue were assessed by the Durbin-Watson statistic at 1.956, 2.049, and 1.909, respectively. A visual inspection of the plot of studentized residuals vs. unstandardized predicted values showed evidence of homoscedasticity for the three regressions. There was no evidence of multicollinearity, as assessed by tolerance values greater than 0.1. There were no studentized deleted residuals greater than ± 3 standard deviations, no leverage values greater than 0.2, and no values above 1 for Cook’s distance for the three types of fatigue. The assumption of normality was met for the three regressions, as assessed by the QQ plots. The multiple regression model predicted statistical significance for all participants [*F*(4, 1,613) = 13.559, *p* < 0.0005], desk work only participants [*F*(6, 935) = 6.697, *p* < 0.0005], and total fatigue [*F*(4, 671) = 9.922, *p* < 0.0005]. All variables presented in Table 2 added statistical significance to the prediction including age and sex parameters (*p* < 0.01).

The results of the statistical analyses show that three, five, and three gray matter regions were significantly correlated with the fatigue scores of the analysis of all participants, only desk work participants, and only non-desk work participants, respectively (Table 2). Figure 2 shows the brain regions that significantly correlate with fatigue scores. Details of the results are given in the discussion section.

## Discussion

This study investigated whether there was a correlation between rGMV and fatigue measured using CFQ scores. In addition, participants were grouped into two groups, desk and non-desk workgroups, to investigate whether different work types affect rGMV or fatigue (CFQ) scores differently.

As aforementioned, there was no significant difference in CFQ scores between the desk work and non-desk work groups. However, there was a significant difference between the two in some rGMVs (right entorhinal area, right middle frontal gyrus, left middle frontal gyrus, and left precentral gyrus medial segment). These differences suggest that the progression of fatigue in different work types affects different parts of the brain, even though participants in both groups did have a significant difference in fatigue scores. Cross-sectional studies cannot explain the causes and effects and/or progression of fatigue, so further longitudinal studies are needed to confirm this possibility.

Most recently, a large study of healthy young students at Tohoku University did not report a correlation between rGMV and fatigue (Nakagawa et al., 2016). Based on the results of the regression analysis shown in Table 2, the present study found that there are several statistically significant rGMVs that can be used to predict CFQ scores. This phenomenon may be because our study examined healthy middle-aged individuals (aged from 45 to 65) who are more likely to experience fatigue compared to younger participants. The affected regions across participants are the left basal forebrain, the right supplementary motor area (SMC), and the right superior parietal lobule (SPL). The right SMC was negatively correlated with the CFQ score; this finding is consistent with previously reported studies by other researchers on CFS patients and multiple sclerosis (MS) patients (Filippi et al., 2002). Thus, it can be assumed that the development of fatigue in healthy individuals has a negative impact on their physical ability if rGMVs reflect the magnitude of neuronal functions among GM regions. The left basal forebrain and right SPL have a statistically significant positive correlation with the CFQ score in all participant data. The basal forebrain and SPL are both known to be related to memory (Damasio et al., 1985; Fujii et al., 2002; Hall et al., 2008; Koenigs et al., 2009; Lester and Dassonville, 2014). A lesion in the basal forebrain can lead to amnesia (Damasio et al., 1985), and it has been suggested that atrophy in the basal forebrain is a presymptomatic marker for Alzheimer’s disease (Hall et al., 2008). The SPL lesion is associated with deficits in the manipulation and rearrangement of information within working memory (Koenigs et al., 2009) and visual short-term memory (Lester and Dassonville, 2014). A positive correlation with the CFQ score means that the higher the CFQ score, the bigger the volume of the related rGMVs. The result can also be seen as a compensatory biological defense response for an early stage of fatigue development in healthy middle-aged adults.

Furthermore, we considered the effect of working type as well as the age range on fatigue development because sitting time significantly has a strong association with morbidity and mortality rates (Gilson et al., 2011). Thus, it was assumed that the longer the sitting time, the higher the fatigue. Linear regression results (Table 2) interestingly show that the rGMVs that significantly correlated with the CFQ score for desk work and non-desk work are different.

In the desk workgroup, it was revealed that the left frontal operculum (FO), right occipital pole (OP), and right SMC were negatively correlated with the CFQ score while the right putamen and left fusiform gyrus were positively correlated with the CFQ score. FO has previously reported a negative correlation with fatigue in multiple sclerosis (Filippi et al., 2002) and traumatic brain injury patients (Berginström et al., 2018). A functional MRI (fMRI) study examining network-to-cluster functional connectivity showed a negative association with the right OP and degree of fatigue in healthy individuals (Boissoneault et al., 2018). As mentioned in the introduction, DTI showed a positive correlation of the right putamen with the degree of fatigue in healthy individuals (Nakagawa et al., 2016).

Notably, in the non-desk work group, the left anterior insula was positively correlated while the right anterior insula has a negative correlation with the CFQ score. The left anterior insula is reported to be associated with maintaining emotional feelings in working memory (Smith et al., 2017), and the right anterior insula may play an important role in maintaining or raising attention ability (Nelson et al., 2010; Gu et al., 2013; Perri et al., 2018). The anterior insula may be involved in regulating fatigue development because of the opposite relation between left and right.

Our results conformed with those of previous studies. In particular, a simple volumetric study using a conventional 1.5 T MRI without using fMRI and DTI enabled the investigation of the relationship between the brain and fatigue. This finding may indicate that data from large numbers of healthy participants from various medical facilities can be used as material for research instead of using the more complex and more expensive process of fMRI. The results also suggest that non-desk work may be reflected more in the motor system in the brain, while desk work may be reflected more in the sensory system. Thus, the two work types may have yielded different mechanisms in fatigue development because the correlated GM regions were all different.

Our study has some limitations. The CFQ score, which was used widely in fatigue examination, did not yield an obvious difference in the degree of fatigue between the desk and non-desk workgroups (Figure 2). Therefore, adding a more detailed category of fatigue, such as physical and mental fatigue, may be required to investigate the difference between desk and non-desk workgroups. Furthermore, alpha-amylase activity in the salivary gland (Michael et al., 2012) or autonomic nerve activity can be used as metrics for fatigue evaluation in addition to the CFQ score. Another limitation was that our study was not longitudinal but cross-sectional, targeting the correlation of the degree of fatigue with rGMV. Additionally, a cross-sectional study cannot explain the causal relation between the fatigue score and the rGMV. It is known that the primary limitation of the cross-sectional study design is that because the exposure and outcome are simultaneously assessed, there is generally no evidence of a temporal relationship between exposure and outcome. Without longitudinal data, it is not possible to establish a true cause-and-effect relationship. Therefore, we plan to conduct longitudinal data analysis to verify these results in a future study. Preparation for collecting data for this longitudinal study is underway. Over the years, we have obtained a large number of potential participants in brain health checkups as a part of regular health inspections. Through a longitudinal study, we expect to find out a more thorough relationship between fatigue and rGMV, ultimately understanding how fatigue progressed from the early stages of fatigue and investigating the mechanism of chronicity. The results of this future study are expected to not only prevent *karoshi* but also establish an effective measure for *karoshi* in order to reduce socioeconomic loss due to fatigue.

## Data availability statement

After publication, by contacting the corresponding author, data will be available to any researcher who provides a methodogically sound study proposal that is approved by the central study team. Individual participants will not be identifiable in any released data and all appropriate information governance.

## Ethics statement

The studies involving human participants were reviewed and approved by the Kochi University of Technology. The patients/participants provided their written informed consent to participate in this study.

## Author contributions

HP conducted all of the analyses, made the figures and tables, and wrote the main manuscript text. KP made a research design, recruited all participants, collected all data, and wrote manuscript text. FY measured the Brain structure data. YN and TM provided insight on statistical analyses and logical configurations. All authors contributed to the article and approved the submitted version.

## Footnotes

1
http://dbm.neuro.uni-jena.de/vbm8/

2
https://www.fil.ion.ucl.ac.uk/spm/
